# A Novel Hybrid Trustworthy Decentralized Authentication and Data Preservation Model for Digital Healthcare IoT Based CPS

**DOI:** 10.3390/s22041448

**Published:** 2022-02-13

**Authors:** Mohammed Amin Almaiah, Fahima Hajjej, Aitizaz Ali, Muhammad Fermi Pasha, Omar Almomani

**Affiliations:** 1 Department of Computer Networks and Communications, College of Computer Sciences and Information Technology, King Faisal University, Al-Ahsa 31982, Saudi Arabia; 2 Department of Information Systems, College of Computer and Information Sciences, Princess Nourah bint Abdulrahman University, P.O. Box 84428, Riyadh 11671, Saudi Arabia; fshajjej@pnu.edu.sa; 3 School of Information Technology, Monash University, Sunway City, Subang Jaya 47500, Malaysia; aitizaz.ali@monash.edu (A.A.); muhammad1.fermipasha@monash.edu (M.F.P.); 4 Computer Network and Information Systems Department, The World Islamic Sciences and Education University, Amman 11947, Jordan; omar.almomani@wise.edu.jo

**Keywords:** cryptography, data integrity, IoT-based-CPS, data privacy, device to device authentication, digital healthcare, IoT, security

## Abstract

Digital healthcare is a composite infrastructure of networking entities that includes the Internet of Medical Things (IoMT)-based Cyber-Physical Systems (CPS), base stations, services provider, and other concerned components. In the recent decade, it has been noted that the demand for this emerging technology is gradually increased with cost-effective results. Although this technology offers extraordinary results, but at the same time, it also offers multifarious security perils that need to be handled effectively to preserve the trust among all engaged stakeholders. For this, the literature proposes several authentications and data preservation schemes, but somehow they fail to tackle this issue with effectual results. Keeping in view, these constraints, in this paper, we proposed a lightweight authentication and data preservation scheme for IoT based-CPS utilizing deep learning (DL) to facilitate decentralized authentication among legal devices. With decentralized authentication, we have depreciated the validation latency among pairing devices followed by improved communication statistics. Moreover, the experimental results were compared with the benchmark models to acknowledge the significance of our model. During the evaluation phase, the proposed model reveals incredible advancement in terms of comparative parameters in comparison with benchmark models.

## 1. Introduction

IoMT-based patient wearable devices and gadgets are employed in an open atmosphere with a radio communication infrastructure, which puts them a risk of new security threats. It is not only patient wearable devices that are susceptible to security risks, but their collected and transmitted data are also at risk during the communication process; therefore, the whole ecosystem needs to be shielded against internal and external threats [1]. In these networks, patient wearable devices act as edges and gateways for end-side data collection and transmission. Therefore, an adversary can easily target and compromise them to extract data from the network or inject malicious data into the network [2,3].

**Figure 1:**
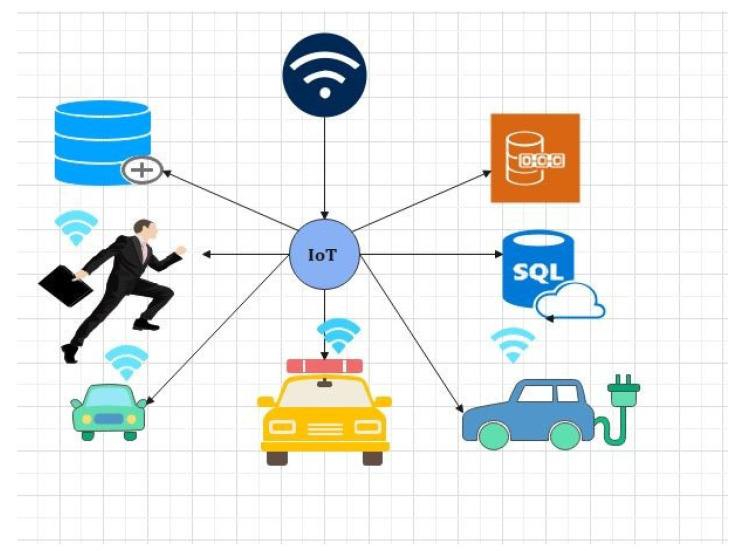
Industrial IoT and its applications in real life.

According to Tractia, an intelligent organization, annual earnings in the sector using blockchain technologies will reach 19.9 billion by 2025 [1]. IoTs are progressively active in health care to give real-time services to patients and physicians [2]. This is accomplished by incorporating IoMT medical devices into medical institutions and businesses. However, as the number of Internet-connected medical devices (IoMT) grows, a greater volume of inconsistent data will be generated [3]. The current implementation aspect of the (CCS) mode, which includes all activities carried out in the center, has significant challenges such as a high latency (HL), network dependency, individual points of failure and failure impact, and an inability to adapt to instantaneous transactions [4]. As a result, the fog or edge computing (EC) prototype supports the time and resource efficient services at the network’s edge. The fog layer (FL) serves as a middleware for management between the edge and the Cloud. Figure 1 shows the basic fog IoMT model [5]. Figure 1 represents the applications of IIOT in various domains. These domains range from smart transportation, smart industries, smart homes, and smart healthcare systems to smart education, etc. It assists the planner in establishing and delivering a service; hence, improving the resource balance and service latency. The 5G network enables mobile networks to connect and control machines and other things [6].

In contrast to these networks, cybersecurity is a weakened realm in traditional computing systems to contend various malicious threats and the infiltration of adversaries [7]. Most of the present cybersecurity explications use simple or complex authentication models, which in the simple cases are susceptible to security threats, while the complex model demands an ample amount of network resources that degrades its real implementation. In-flight IoT drone security and surveillance are a few examples of 5G-enabled IoMT communication applications. These apps help the community every day. Everything in this ecosystem uses the Internet. So, this message requires security and privacy [7]. It also faces other threats. Defending 5G-enabled IoT communication infrastructure against these threats is crucial. These protocols include key management, user authentication/device authentication, access control/user access control, and intrusion detection. This paper details many 5G-enabled IoT communication system models (network and threat models) with the communication environment’s security dangers and requirements. Moreover, the security techniques for 5G-enabled IoT connectivities are compared. This research has been used in various fields, including the streaming of the 5G-enabled IoMT and the development of safe, secure medical data transfer methods. The Cloud with a blockchain-based fog architecture (FA) provides a bigger foundation for coordinating remote resources and completing jobs [7]. Unlike traditional Cloud computing (TCC), the hybrid service environment, which incorporates the edge or a secure IoMT layer that services blockchain [8] has more unpredictable and diverse uses, necessitating a more flexible, resilient management by using the proposed method. We designed a data privacy technique to keep private data protected during DL training and inferences for fog computing. The data preserving technique presented in this paper is designed for preserving EMR used to distinguish authentic and valid users from intruders and hackers [9]. The DL model does not have access to any identifiable information. It takes only encrypted images as inputs. The encryption of the chest X-ray is completed from the user side before it is fed to the DL model. This encryption is homomorphic, which means that the final classification result does not need any decryption of the image. The DL model will only work with these encrypted data without requiring any personally identifiable information. We think this feature is essential, especially in the healthcare system using fog-computing. In fact, several research works have been conducted to show that EMR datasets are important nowadays because of their privacy. Moreover, many people explicitly require that the body details shown in these kinds of images be kept private and secure [10]. The technique presented in this paper fulfills this requirement at a very low cost (around a 1% decrease in accuracy). We explain the detail of hybrid Deep Learning techniques for privacy using homomorphic encryption.

In response to these challenges, in this paper, we propose a hybrid lightweight authentication scheme, which is basically designed from two different attributes such as the supervised machine learning (SML) technique and the Cryptographic Parameter Based Encryption and Decryption (CPBED) model to ensure the validation of legal devices followed by secure data transmission in the IoMT-based CPS. In this scheme, the authentication of legitimate patient wearable gadgets is enabled in a decentralized fashion by exercising the SML technique to predicate the nearest authentication server (AS) and radius server or service provider (SP) to validate communicating devices by managing public and private keys. The SML algorithm allows the patient wearable devices to use a hop count communication infrastructure with proper validation in a decentralized atmosphere to depreciate the overheads and complexity of the network. Unlike ordinary authentication schemes, the intended prototype uses multiple AS SP to assure the validation of patient wearable devices in smart healthcare such as IoMT-based CPS, where multiple hospitals are interconnected through the Internet.

Recently, several research works related to data privacy protection and the identification of cyber-attacks, particularly using the Cloud and edge computing integrated with blockchain, have been proposed to tackle the above issues. However, it should be noted that there are still various drawbacks in the system [11]. The major challenges are constructing a privacy-preservation scheme to protect the sensitive data transactions from being accessed by unauthorized users [12]. Moreover, ensuring a secured authentication data transfer scheme, and maintaining data integrity when communicating the data over IoT network is a challenging issue. Second, designing an adaptable security mechanism that can efficiently distinguish between normal and attack instances in IIoT is also a challenging issue. As such, the IoT network comprises various interconnected medical sensors, actuators, and machines (e.g., VMs and platforms), located at multiple locations [13]. Third, developing a new framework for deploying blockchain and deep-learning techniques in current Cloud-edge-assisted industrial systems is strenuous. As such, the framework often faces issues related to scalability; moreover, due to the different computing powers of the participating edge nodes, it is infeasible to store the complete block in the edge networks [14]. Figure 1 describes the application of IoT applications in real time scenarios. Therefore, it can be easily observed from Figure 1 that the number of applications is too large using IoT and IoMT techniques.

### Key Contribution

In this paper, motivated by the above challenges, we designed and implemented a blockchain-based deep-learning framework to enhance security and privacy in IIoT.

The key contributions of this paper are as follows:

1. We propose a Proof of Improved consensus algorithm designed for blockchain to validate the blocks before they are committed to the ledger;
2. The design of a deep learning-based secure model to identify honest miners and restrict malicious miners;
3. We present a complete working solution for the integration of the proposed consensus algorithm with the Ethereum Framework;
4. A comparative analysis of the existing consensus and the proposed consensus protocol is presented;
5. The design of a novel algorithm is added in order to secure the proposed model.

## 2. Background and Related Studies

Smart healthcare, which is constituted from patient wearable IoT devices, encompasses various security vulnerabilities and susceptibilities, i.e., the authentication of patient wearable devices, data privacy, and preservation, etc. [14,15,16]. Authentication of multiple wearable devices in a network is a critical part of secure communication because it guarantees the users’ identities. Even though many experts have worked in this field to alleviate the known vulnerabilities and threats, over time these authentication models become susceptible to external and internal threats as the adversaries continually try to tamper and hijack them. Therefore, this domain is still open to new authentication schemes that could help to promote the validation process with better communication attributes. In [17], a community identity-based authentication model was proposed for healthcare IoT networks to address the security concerns in these networks. In this model, an elliptical curve cryptographic (ECC) was performed primarily to produce erratic bio-cryptographic signals to assure the validation of legal devices with secure communication. The authors of [18], presented a certificate-less-based authentication model for wireless body area networks to fix the security solicitudes of these networks. The storage of patient health records over the Cloud provides various opportunities and challenges. Cloud-based access control models are more susceptible to security breaches and a secure access control system is needed for current PHR-based models, since a Cloud-based framework mostly works in an open and integrated environment. Due to these features, Cloud-based networks are more vulnerable to data loss, theft, and security attacks. A weak network security system is one of the most highlighted and explored problems which has directed IT researchers to explore more smart security directions and tools for the Cloud using medical health-related data. The digital healthcare industry has many reasons not to trust the Cloud environment, because it cannot provide complete access control to patient health records. The fog computing-based IoMT is currently a hot topic [7]. Previous research works have missed important security issues such as:

1. Healthcare IoMT devices send data to Cloud servers that are frequently unencrypted and open to manipulation and attack. As a result, sensitive patient information will likely be accessible.
2. To our knowledge, the need to identify IoMT medical devices, which leads to the verification and authentication of health data, is urgent, and it can be accomplished quickly using a blockchain in the FC-IoMT system. Servers at the network’s edge should perform more detailed authentication and verification.
3. BAKMP-IoMT, the new IoMT key agreement technique for blockchain-accessible authentication, was designed by Aazam et al. [13].

Between the medical devices implanted and the personal server, BAKMP-IoMT provides a secure key management mechanism. P. Gope et al. [16] presented a revolutionary anonymous Internet of Things authentication mechanism resistant to machine PUF attacks. Salem et al. [14] developed a strategy to avoid interference with MitMs and prevent alarms from the remote health surveillance system. P. Zhang et al. [15] used a profound learning model with the deep convolutions neuronal network (CNN) and a short-term long-range memory network. The approach described by Z. Ning et al. [16] can achieve a Nash balance. It is also obtained theoretically from the algorithm’s top time complexity and the number of MEC patients. Mobile-based healthcare system was proposed by Liang et al. [17]. This is also called a record sharing framework using BC through an approach based on user-centric security to limit the access of unprivileged users and to enhance the privacy via a channel formation scheme. The issue in this approach is the computational cost due to the complex cryptographic mechanism. However, using a health technology blockchain, Dwivedi et al. developed a peer-to-peer strategy for linking distant medical sensors and equipment through the Internet. They came up with the notion of a better blockchain foundation for IoT devices. In a decentralized context, the suggested approach by these authors provides higher security for a healthcare system.

Dwivedi et al. proposed a peer-to-peer approach based on a privacy-preserving digital health blockchain for connecting remotely medical sensors and devices. They proposed the idea of an improved blockchain framework appropriate for IoT devices. This proposed framework of these authors works in a distributed environment to provide more privacy to a clinical system. Aggarwal S. et al. investigated several outstanding research topics on readers’ 5G-enabled Tactile Internet fog computing. This is also something that researchers have investigated. Ahad A et al. thoroughly examined 5G-assisted smart healthcare solutions in the IoT. R. Cao et al. proposed a multi-Cloud cascade architecture, and a low-overhead native testing framework anda medical data storage backup method was proposed by B. D. Deepak et al. A smart service authentication (SSA) system was proposed to improve patient–physician data security.

The impact of the increased security vulnerability of electronic systems is exacerbated for devices that are part of the critical infrastructure or those used in military applications, where the likelihood of being targeted is very high.

In addition, a systematic threat modeling analysis and security validation is included in this paper, which indicates that the proposed solution provides better protection against information leakage, loss of data, and the disruption of operations. The rest of the paper is organized as follows. Section 2 discusses various privacy-preservation and intrusion detection techniques, and relevant existing work. In Section 3 and Section 4, we present the proposed framework with its various functional components, and our evaluation approach. Finally, the paper is concluded in Section 5.

### 2.1. Preliminaries

The following concepts are used in this research paper and are explained below.

#### Blockchain-Based Fog Computing

**Figure 2:**
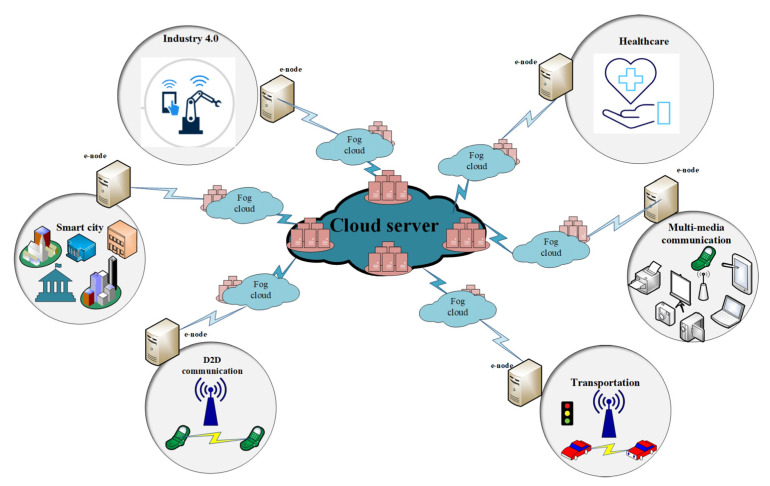
Applications of fog computing.

A blockchain and fog network [18,19,20,21,22,23,24] connects the Internet of Medical Things (IoMT) and fog nodes (FN) (IoMT-fog). Distributed technology can deliver on-demand services by combining high performance and low latency (LL). It will raise the threshold for monitoring people’s health. The FC paradigm aids IoMT elements with a low latency (LL), allowing for faster data processing. The proposed IoMTfog, shown in Figure 2, could provide a more appropriate medical equipment (ME) solution [18].

### 2.2. Proof of Work

PoW is a blockchain consensus that requires network participants to solve a random mathematical puzzle. In this mechanism, a group of individuals compete against one another to complete a transaction on the network; this is defined as mining. One of these miners with a high computational power will solve the cryptographic puzzle and be considered the winner. The miner creates the new block and receives a reward. The ethereum network is based on the PoW consensus for making a decision and resisting attacks. In Figure 2 we have explained the proof of work concept for fog-based computing in the IoT environment.

#### Limitations of PoW

The first is that electricity waste is a critical issue in the blockchain environment. Peers consume additional energy when performing computation work. Second, a 51 percent attack is a severe challenge in a small network. A 51% attack occurs on PoW when a group of attackers with high computation power or hash rate control the entire network. Because of the high computation requirement, mining pools may govern the mining game, resulting in centralization and vulnerabilities [19]. PoW tends to centralize and over time several people wind up with all the resources by the computers. They capitalize on the scale of the economy once they have a mining form, which makes it cheaper to obtain the electricity and buy the machines needed to stay in the control of the network [20]. When it comes to non-financial use cases, PoW has a problem in that the thing we are trying to transfer becomes more valuable than the electricity.

## 3. Proposed Framework

The description of the proposed framework is provided in three parts, which are provided as below. The systematic architecture of the proposed framework consists of three layers, namely:

1. IIoMT-enabled industrial layer;
2. Edge and fogblockchain layer;
3. Cloud–blockchain layer.

As illustrated in Figure 1:

1. **IIoMT-enabled industrial layer:** This layer consists of IoT-based industrial track-ing systems and implantable medical devices (e.g., glucose monitor, temperature sensors, heart rate devices);
2. **Edge–Blockchain layer:** This layer consists of powerful nodes, i.e., full nodes (FN), that include edge-computing servers, industrial computers, data analysis servers, and so on. Here, the peer-to-peer network is created by the edge devices situated at main and urban health centers through geo-distributed areas. Every patient is enrolled to an edge service node which is responsible for gathering patient data, processing it, raising alarms in emergency scenarios, and communicating with the Cloud for backup and long-term storage [22,23,24,25,26,27,28];
3. **Cloud–Blockchain layer:** Various Cloud suppliers and data centers are included in this layer. These data centers (DC) are responsible for providing services (such as processing, calculation, and so on) to clients. An IoT-enabled industrial system mainly consists of LNs, FNs, and DCs. The LNs have resource constraints, and therefore they can send the data to FNs in the edge–blockchain layer. The FNs can assist LNs to search transactions, and can be used for mining, and adding a new block in the blockchain network. Finally, DCs are responsible for the long-term storage of data from FNs as required [29,30,31,32,33,34,35].

**Figure 3:**
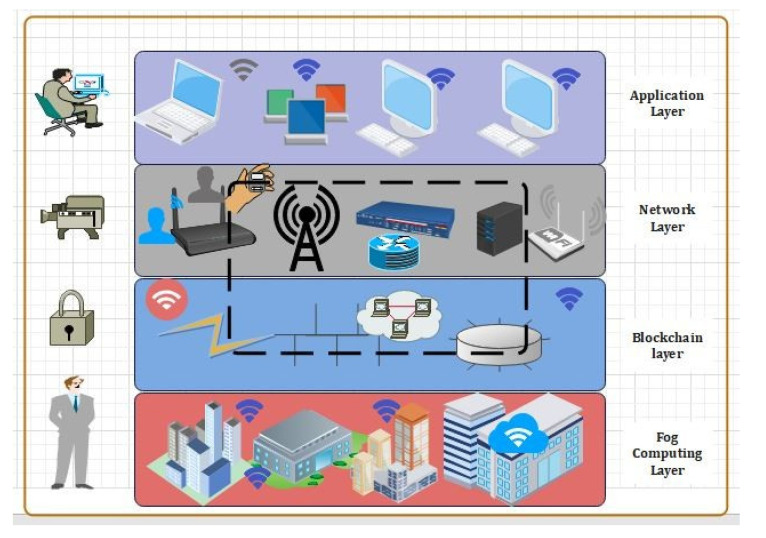
The proposed secure searchable encryption system and the system layers.

Th proposed framework is shown in Figure 3. These devices constantly capture the patients’ critical health parameters and due to limited resources and computing power, these devices can store and process part of data on the blockchain and are termed lightweight nodes (LN). Figure 3 represents our proposed secure searchable encryption system and the layout of the proposed communication. Figure 3 provides a detailed insight into the structure of the three layers that we propose for our framework. Each layer has its own function and responsibilities as described through Figure 3. The DCs send data to the edge as per the requirement. The proposed blockchain-based security and privacy scheme is used to register all three nodes, and therefore, authenticates the data transactions in the network using proposed smart contract-based ePoW. In addition, the IPFS storage system is used to store complete transactions and the generated hash string is stored in the blockchain. Finally, the DL-based privacy and security scheme is used to transform and detect intrusions in the network. This scheme is deployed as Software- as-a-Service (SaaS) at various network nodes (i.e., routers, gateways, edge servers, and Cloud data centers). Further, the framework is deployed in a large-scale distributed network model or an individual host that makes communications successfully, either in the edge–blockchain and the Cloud–blockchain layer, and further it coordinates with others to detectcyber-attacks [36,37,38,39,40,41].

### 3.1. The 5G Technology and Its Applications

The network deployment of 5G and the marketing research began in 2014 and is expected to be finished by 2021. In addition to network densification and support for a wide range of IoMT devices, 5G [10] networks should enable higher data rates (DR). The 5G networks are designed to support intelligent IoMT-based medical applications that require high data throughput, scalability, blockchain rollout, low latency, dense deployment, reliability, high energy efficiency, and long-term communication. Figure 3 shows the fogIoMT Architecture for the security of IoT device-based healthcare records using blockchain technology [42,43,44].

#### 3.1.1. Fog Computing (FC)

**Figure 4:**
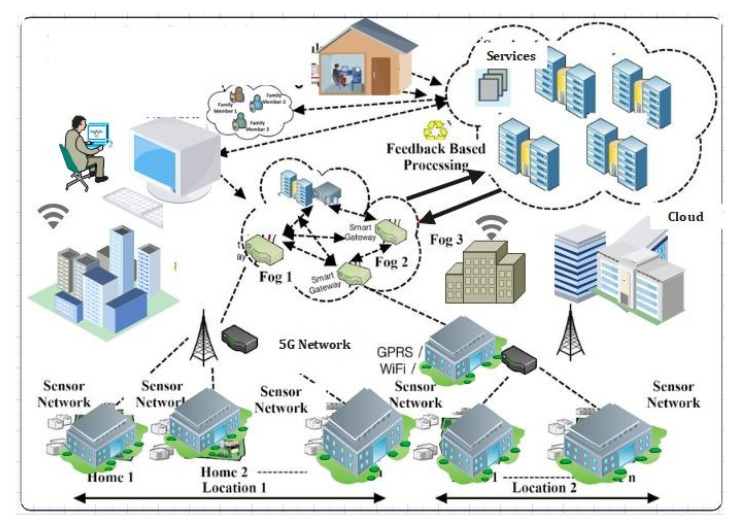
Proposed secure searchable encryption system using a DL-based intrusion detection system.

Recent advances in fog computing and fog-Internet of Things (fog-IoT) technology involve data analysis and AI-based medical operations. This model’s main flaw is its susceptibility to security threats and cyber-attacks even against fog computing layers. In this scenario, each layer is vulnerable to attack, including the edge layer (sensing), the fog layer (processing), and the public layer (storage and management) (Cloud). Fog-Internet of Things (fog-IoT) is currently and broadly the backbone of intelligent health care systems. The proposed blockchain-based fog computing using 5G technology is illustrated in Figure 4. Figure 4 completely describes the flow of data via the fog-based blockchain using a high data rate Internet called 5G Technology. The fog-IoT is designed to overcome the limitations of the secure data access (SDA), data storage (DS), and scalability associated with IoT medical devices [21]. However, the rapid growth of IoT-based medical devices and managing such a broad, sophisticated medical IoT system on standard Single Cloud platforms (CP) would be extremely difficult. We propose a scalable FC with blockchain-based architecture for the 5G-enabled IoMT platform. To work on an FC architecture with flowing effects, low overheads, and secure storage (SS), this research proposes a secured blockchain-based fog-BMIoMT communication mechanism [22].

#### 3.1.2. Blockchain-Enabled Security and Privacy Scheme

Registration Phase: In this phase, the registration of the data center (*D_C_*), full node (*Np*) is completed securely in the off-line mode by a trusted registration authority. In addition, the light node, i.e., the sensor node (*NS*) is registered with (*Np*)using the zero knowledge proof protocol (KPP). This protocol authenticates two parties without revealing any secret identity or information. In this approach, one party becomes the challenger and the other party becomes the prover. If the prover response is correct, then it becomes the verified party. Here, (*Np*) registers (*Np*) by initiating a request after zero knowledge proof verification. The steps of registration and verification process are discussed below:

In the initial step (*Np*)generates a temporary key (*kt*) of (*NS*) which consists of three major parameters: (i) *sensor* temporary identification of (*i_t_*); (ii) *MAC of sensor*.

#### 3.1.3. Proposed Neural Network

**Figure 5:**
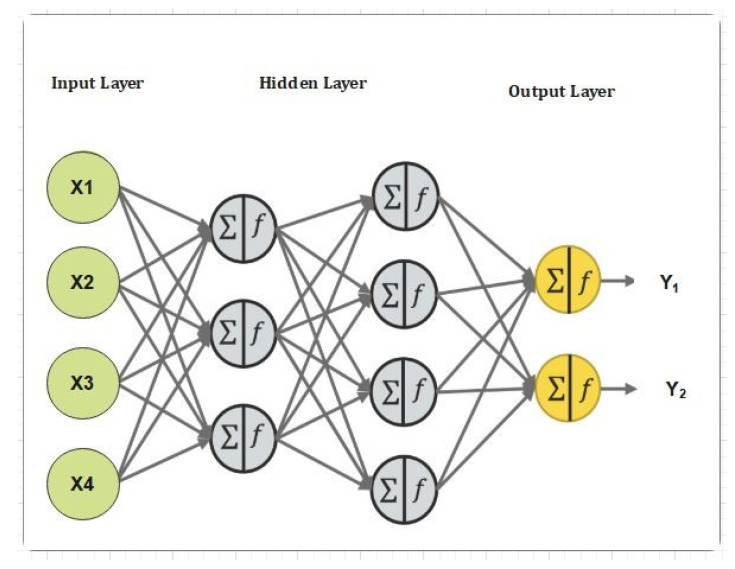
The proposed Neural Network system for fog-computing.

**Figure 6:**
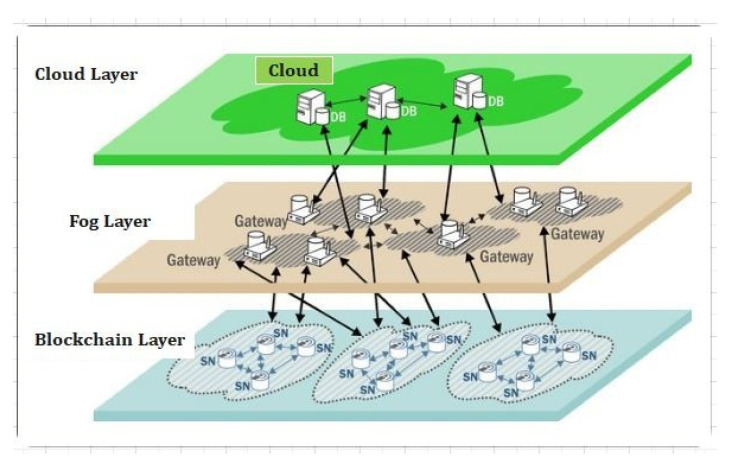
Proposed secure searchable encryption system using a DL-based intrusion detection system.

**Figure 7:**
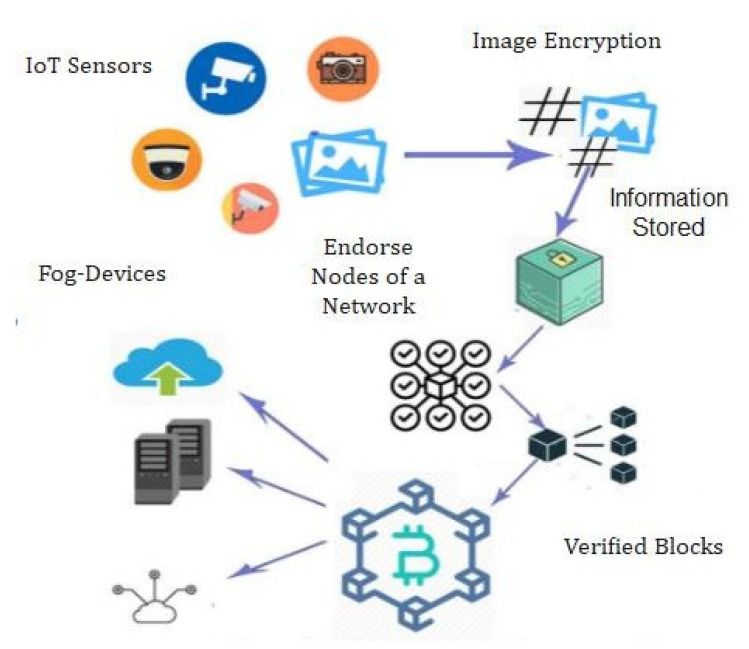
Proposed Neural Network system for fog-computing.

Our proposed neural network system is shown in Figure 5, Figure 6 and Figure 7, respectively in complete detail. The proposed neural network consists of input layers, hidden layers, and an output layer. In Figure 5, the proposed neural network model is presented. Figure 5 also explains the number of hidden layers, input layers, and output layers. The complexity of the proposed neural network can be seen in the number of hidden layers. The more hidden layers, the more complex the system. Figure 6 represents the detailed structure of our proposed convolutional neural network (CNN) and shows how we trained the dataset. We used an IoT-based dataset and we divided it into two parts. The division of the dataset was based on the testing and training. We trained 30% of the data and then we tested 70% of the data. Figure 6 describes each step carried out through the training and testing in a schematic way.

#### 3.1.4. Proposed System Architecture

**Figure 8:**
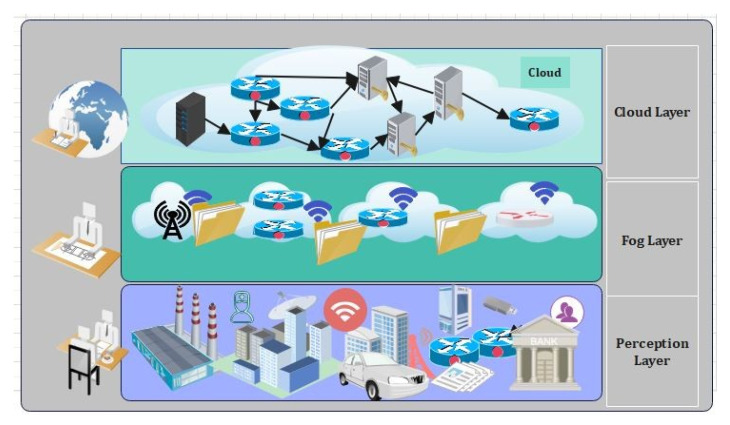
Proposed secure searchable encryption system using a DL-based intrusion detection system.

Several physical servers combined in an FN configuration to cover a specific diameter region. The fog nodes (FN) can be wired or wirelessly connected [23,24,25]. The FN provides processors and sets up the equipment and network services as a small virtual data center. The processors, software, and network services made up the fog nodes (FN). Smart sensors (SS) collected data from the surrounding environment, which FN analyze and uses to understand decision-making better. The FN also provides a 5G network with a limited range of unicast wireless connections. Concurrent data transfer is enabled by the newly created 5G network protocols, which allow packets to be sent to all or a selected destination simultaneously [26]. The FN, a storage system for passive memory resident programs, can include the local database. For heavy IoMT applications, this means faster processing and loading times [27]. The Internet of Things in the Medical Field (IoMT) is a network safety, reliability, bandwidth, and optimization application that affects IoMIs, IoMT, and IoMT network safety deployment, reliability, bandwidth, and latency optimism. In this context, several FNs provide dynamic information as represented in Figure 8.

In this research work, we considered a series of hospitals as a case study, where several inpatient wearable CPS, authentication servers, and base stations are interconnected. In a hospital, there are several units i.e., gastrology, cardiology, pulmonology, hematology, pathology, radiology, wards, patient reception centers, discharge lounges, and an emergency transportation center followed by private rooms to facilitate patients. These units, wards, and facilitation centers are interconnected to access, assess, store, and transmit the patient’s data accompanied by other important credentials in the network. As shown in Figure 7, an AS employs the prognosticated information of the SML technique to contrive the validation process of legal patient wearable devices within its proximity region of an individual hospital in a decentralized environment rather than sending it to a particular AS, which manipulates the authentication process in a centralized environment.

The entire Internet layer represents the actual user environment, where programs can be deployed without restriction. IoMT devices are divided into categories based on their location and function. This conserves energy, reduces consumption, lowers prices, and saves time. Software and hardware services help data centers integrate and process information. Peer-to-peer (P2P) TCP/IP can be used to communicate between IoMT devices across short distances [28]. They can use FN via WiFi, ZigBee, and Bluetooth-like technologies if they are remotely apart.

**Figure 9:**
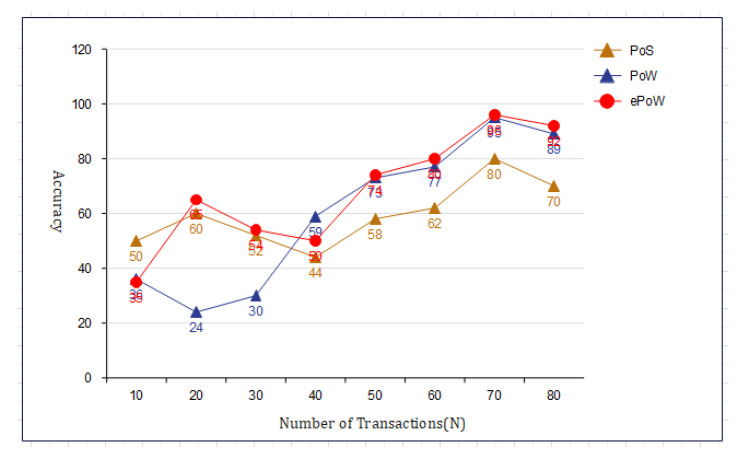
Proposed Neural Network system for fog-computing.

**Figure 10:**
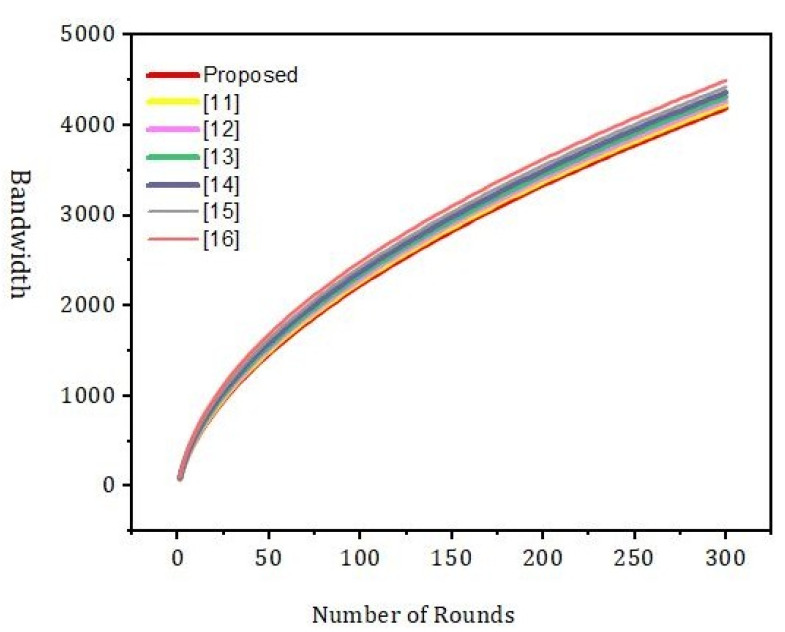
Simulation results based on the bandwidth and number of rounds.

Figure 9 shows the system architecture of the proposed model. The system architecture consists of the blockchain, fog computing devices, and smart contracts. The proposed smart contracts check the access control policy using deep learning techniques and if the user has enough attributes, then access is provided, otherwise access is denied. Figure 10 describes the three-layer structure of the proposed framework. The first layer consists of the perception layer, the second layer consists of the fog layer, and the third layer consists of the Cloud layer. Each layer has its own function, and it supports different devices. Our proposed framework works on these three layers. IoMT and blockchain (BC) use for centralized communication paradigms which are part of the existing IoMT. Centralized Cloud servers will validate IoMT devices. As a result, the current IoMT solutions for sharing health-related data rely on Cloud computing (CC) and network resources for infrastructure and maintenance. A Wireless Body Area Network (WBAN) with devices that constantly expand and drop is deployed in the medical field.

### 3.2. Proposed Algorithm

We have proposed novel algorithms for the proposed framework. The proposed algorithms 1–3 are described below.
**Algorithm 1** Algorithm Transaction Creation and Encryption 
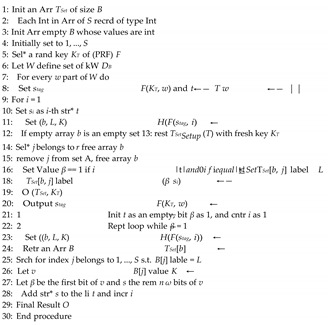

**Algorithm 2** Algorithm Transaction Mining1: Proc Mining Transactions2: Init3: While*ω            τ* then   ≤
4: If *τ*5: Ret T value6: Else7: Ret F Value8: If9: Inc block num10: Else11: Inc is not performed12:  End Procedure
**Algorithm 3** Algorithm Method Evalution1: Enhance Manifold Analysis Evaluation of both the IoMT end2: SelectIoMT device for comm 3: Obtain acquisition, hash, electronic medical records (EMR) 4: Extract EMRFromRepository from EMR (EMR name) 5: EMR, valid SHA checkHash (EMR, hash) 6: if EMR, valid is true, then 7:  Obtain the Connect Length using Connect length (Connect) 8:  Generate Indications(Connect length) Generate Indications(Connect length) 9:  F Blockchain transaction addAnalysis(i, indications) 10: deleteLocalEMR 11: end if (EMR) 12: end 13: end


### 3.3. Dataset

The initial dataset is presented with all the information from the log file described above. Making a split for each space in each log row results in ten columns. After inputting the log file and extrapolating a schema, only the columns needed for future training are selected. In particular, the IP address is essential to differentiate the requests made by client made and then the label is created to differentiate licit users from illicit ones. The date is important to create the 30 s periods necessary for the model to perform the training, for the size of the request bytes, and also for the only one to give input to the neural network. The final dataset is structured in such a way as to have periods of 30 steps, each regarding the sum of requests made by an IP address in a small range of time, with three columns: IP, Byte, and Label; the latter identifies the type of user (licit, illicit). The IP column has not been removed because in order to test data in a real environment, where you will not know if it is a licit or illicit request, once the prediction has been made you will need to identify the attacker in case a DDoS attack is in progress. The information exchange in the target application comprises a large amount of healthcare device data, such as IoMT, which is rapidly growing. There is a need for more bandwidth, data storage, and capacity. It sends data to and from local storage devices, online devices, and the Internet. The data is remediated, filtered, and merged under corporate standards. In this case, the Cloud is used as the final layer for metadata processing. Data and metadata analysis are summarized by FN (edge or dew computing (DC)). With FN and dew computing (DC), the proposed fog-IoMT Architecture improves the mobility of IoE (Internet of everything) users. Blockchain (BC) adds a second layer of security to prevent anonymous users from using IoMT devices. The following is the order in which the communication takes place: (a) Wireless Internet access is used to communicate with IoMT devices across a medium distance. TCP/IP is utilized for inter-primary communication, whereas ZigBee and Bluetooth are used for primary communication. (b) Wireless or wired media communicate between Cloud computing, dew computers, and the fog nodes [30]. TCP/IP end-to-end connections are made via a CAT-5/6 optical fiber. There are two sorts of communication: direct and indirect.

In [22], an anonymous authentication scheme was proposed for wireless body area networks to securely transfer data in the transatlantic communication environment. To address the data privacy preservation, authentication, and integrity solicitudes in healthcare IoT applications, a multi-factor authentication model was proposed in [23]. In this model, the authors used biometric, smart care, and passwords as a combination to enable authentication between the client and server. Consequently, a fine-grained-based authentication model was proposed by Chatterjee et al. [24] to manage the validation of legal IoT devices in a telemedical information system. The authors of [25], presented a software defined-based secure framework for an edge computing-enabled healthcare ecosystem. In this scheme, edge servers were used to verify authorized devices in an operating network. In [26,27], novel authentication schemes were presented for healthcare IoT networks utilizing a blockchain-based infrastructure.

### 3.4. Identification of Threats

Under the above scenario, three main types of threats are relevant: information leakage, tampering, and sabotage. The sabotage threat is not going to be addressed in this work; in practice, this risk can be mitigated, for example by enhancing the physical security of the device deployed, but it cannot be completely removed given the assumption that the adversary has physical access so they can destroy the device or shut it down. A more serious security challenge, in this case, is information leakage. Take, for example, drones that are frequently flown into hostile territories. These devices are very likely to be shot at if discovered, which can cause financial losses. However, if a drone is captured by an adversary, this can have more dire consequences. Extracting information from such a device (e.g., origin, mission, and likely destinations) is much more valuable to the enemy than destroying it. Another threat, in this case, is false alarms, caused by noise or environment variations. The latter may trigger undue tamper response mechanisms such as powering down the system or the deletion of sensitive data, which undermines the system’s availability and disrupts its operation. The identification of threat mechanisms is explained in algorithms 1, 2 and 3. Our proposed algorithm only allows authorized users to access the EMR through fog computing

### 3.5. Modeling of the Analysis Process

The analysis of previously recorded connected network data is the second major phase in the 5G inspection process. We tested the blockchain network in the simulation with various analysts operating at the same time. Algorithm 3 shows how the analytical procedure was coded. Every analysis must analyze Nominalise inputs. To begin, the analysts must retrieve acquisition information from the blockchain, download a copy of the raw data electronic medical records (EMR), and validate the hash value of the EMR. The process obtains the connect length, produces certain indicators, and runs the Add Analysis transaction if the check is successful. Lastly, the raw data EMR’s local copy is erased. For secondary communication, multicasting is supported. With a high-speed connection and low latency, the QoS of inter primary and primary communication increases. Fog-IoMT supports flux/ubiquitous applications, heterogeneity, and secondary connections compared to previous communications systems. Because of the distance between the components, secondary and primary communication is separated. The FNs’ scalability has increased or decreased. The local connection is controlled by primary communication, while secondary communication controls the external connections. A subset of key communication is the T2T relationship. The applications were vetted and authorized on the blockchain. To avoid congestion, a fog-IoMT IoMT layer on a secure channel was utilized. This was also performed to communicate with TFNT.

### 3.6. Attack Risk Factor

The stake value was calculated using the actual cost of various values estimated from exchanges as follows. Where the current value of a token in the staking queue, that is modified every block interval. is the total number of tokens staked during the current block interval and is the number of tokens adequate for mining the transactions. Agreement nodes in PoIP are referred to as miners since they are responsible for validating, confirming, and constructing honest blocks. Miners can invest their original tokens or receive tokens assigned by other existing shareholders. To calculate the security risk of PoIP systems, we assumed that the number of different tokens in the stake pool was the same and added a “generic” token; its price is the total of a token value in the PoIP staking blacklist as mentioned in Equation (6). Where n is number of tokens used for computation in PoIP. represents the value of token i in the PoIP stake blacklist where and. To initiate an attack on the PoIP blockchain system, intruders need to possess a sufficient amount of and hold the majority of the stake value. The probability density function of is calculated as below:

*P*(0 *< ω* ≤ *π*) = ∫(*f* ≤ *λ*)*dy* (1)

The price for the associated security risk element is computed as follows:

*ρ* = *τ*
*≤*
*ω* (2)

*U* = *P^T^X* (3)

The estimated variables and samples are taken as *m* and *n*, respectively. The derived principal features are enclosed as:

*m* = *n* ∗ *U^T^* (4)

and the eigenvectors of the selected covariance matrix are enclosed as:

*m* = *m* ∗ *P^T^* (5)

and can be written as:

*T* = *T* ∗ *X^X^* = *l* ∗ *U^P^/P* (6)

Some of the last eigenvectors are equal to 0 and can be ignored; however, it is ineluctable to sustain more eigenvalues. Then the normalized principal features are shown below:

*S* = *U*^1^/2 = *P^T^X* = *OX* (7)

This can be used for the estimation of independent component (IC) and the *O* can be represented as:

*T* = *P*(1/2) (8)

The main aim of this method is to estimate the matrix:

*m* = *d*/*B^T^* (9)

*B* = *B* ∗ *E*/*T* (10)

*Throughput* = (*Number of Request*)(*Number of ExecutionTimeinseconds*) (11)

## 4. Results and Evaluation

In this section we present the simulation results and their evaluation. As depicted in Figure 9, the values of the three metrics resulting from the training of the model were all very high, which shows that the model reacted well to illicit users but at the same time it didnot exchange the licit users for malicious ones. From these data, the confusion matrix shown in Table 1 was derived. The paper aimed to use the Machine Learning model presented to fight in a more accessible way the type of DDoS attack at application level. The values of accuracy, precision, and recall give an idea of this; it should be used and then adapted to more complex and real systems that acquire much more data, thus creating much more periods, and changing parameters such as the number of steps, the time between one step and another, the number of iterations, and everything that can improve the model according to the system to which it interfaces. The use of the proposed approach involves modeling and studying the infrastructure on which it is to be applied. The data found in this work requires feedback from more impressive infrastructures and, above all, from a realistic system. The categorization of requests in bytes into ranges depending on the types of resources found in the system should be tested. This could further facilitate the learning of the model since it would have categorized values and no longer the normalized values of the requests. Moreover, the solutions taken in action in the model (number of hidden layers, number of features, number of hidden units, number of steps, time ranges, iterations, epochs, batch size, loss function, optimizer, etc.) could be varied.

**Figure 11:**
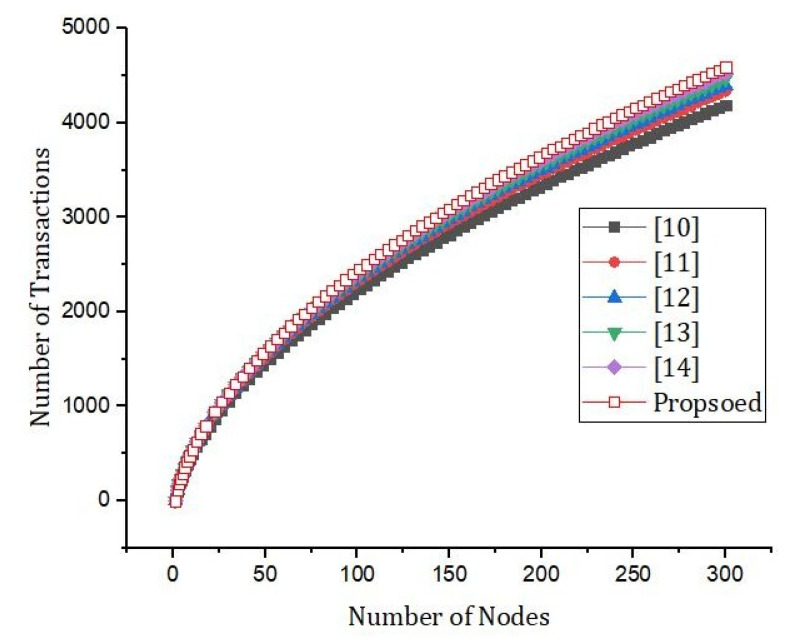
Simulation results based on the bandwidth and number of rounds.

**Figure 12:**
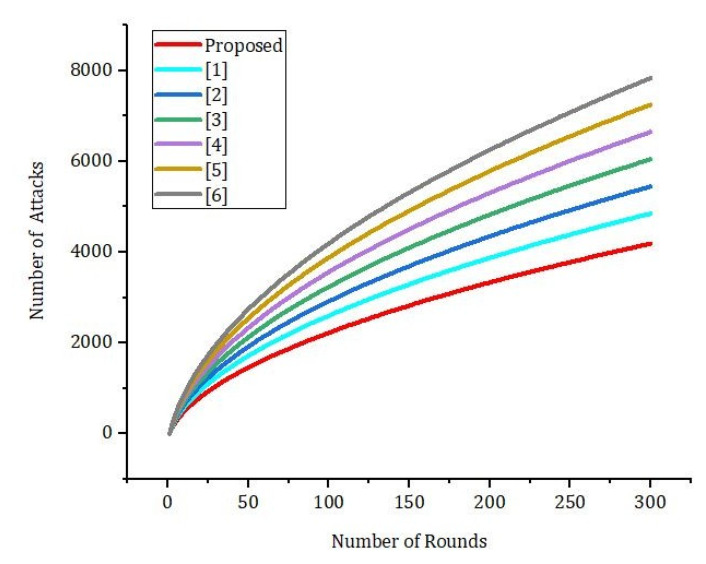
Simulation results based on the bandwidth and number of rounds.

**Figure 13:**
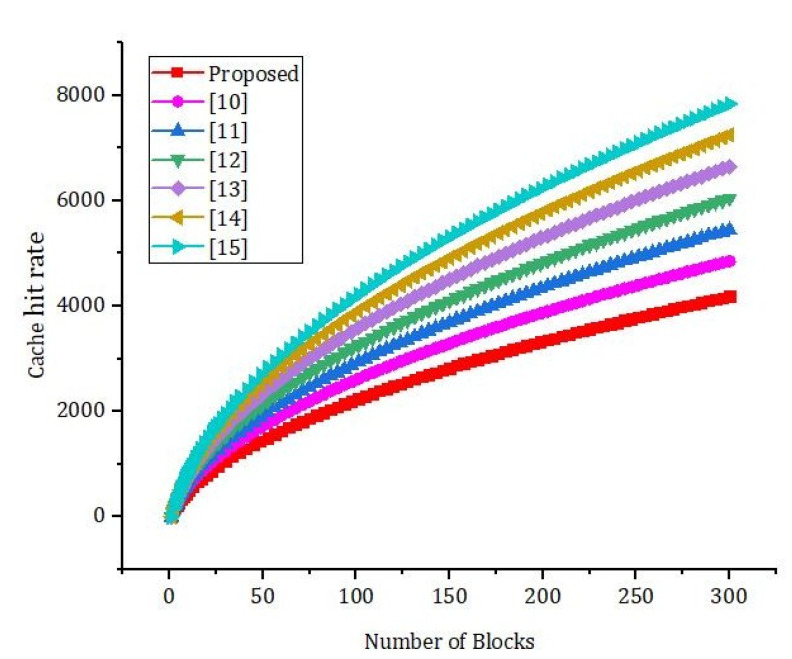
Simulation results based on the bandwidth and number of rounds.

**Figure 14:**
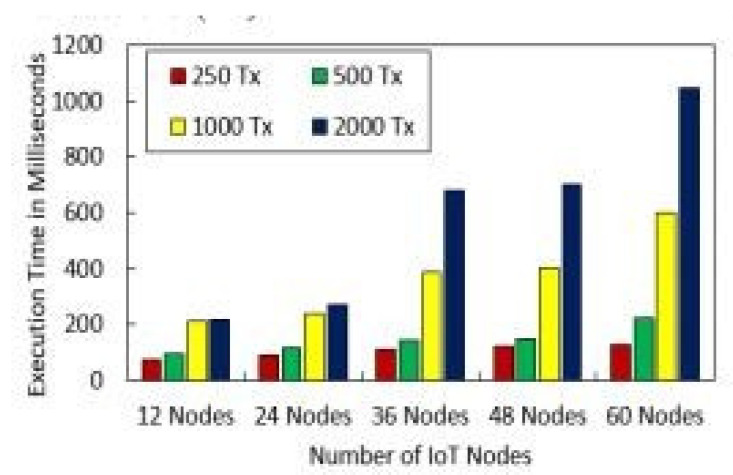
Simulation results based on the bandwidth and number of rounds.

**Figure 15:**
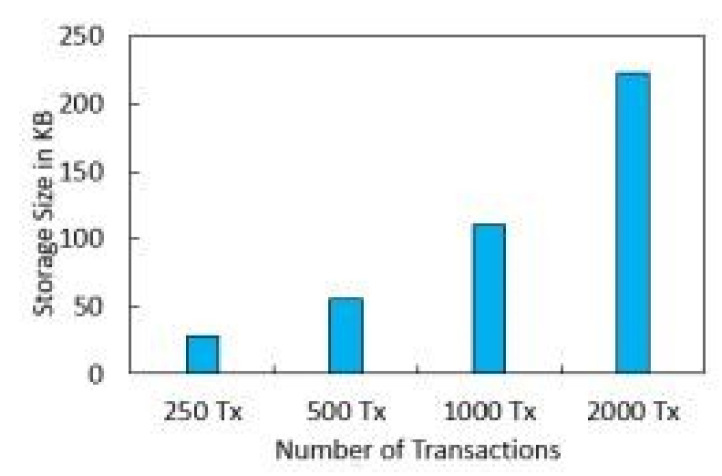
Simulation results based on the bandwidth and number of rounds.

**Figure 16:**
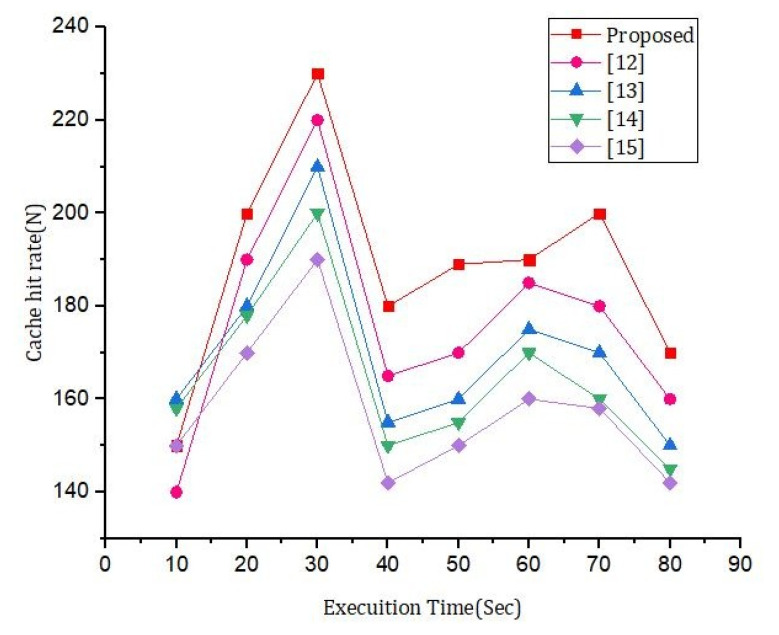
Simulation results based on the execution time and the cache hit rate.

Figure 10 shows the effect of the number of rounds and the error rate. We compared our proposed framework to the models that are already out there. We can see from Figure 11 that our new framework is more efficient than the benchmark models. In Figure 12, we ran simulations based on how many transactions and how accurate they were. In the first simulation, the honest node behavior was set to make real blocks and real votes when the nodes were chosen as miners, while malicious nodes made fake blocks and fake votes together when nodes were chosen as miners. As shown in Figure 13, in this simulation, we changed the percentage of the malicious nodes so that we could see how well the proposed framework worked and how well the benchmark model worked based on the bandwidth and number of rounds. It was set to 0 to 300 nodes. Figure 13 shows how it looked. The accuracy of PoIP was better when there were a lot of nodes, such as 500 or more. As soon as the percentage of malicious nodes is more than 50%, the accuracy will go down a lot. If the proposed framework is better than the others because the positive value is used to pick honest miners, then it is better than the other frameworks. The percentage of a malicious node in real blockchains does not go above 50% in this kind of case. It is important to see how quickly one block creation works so we ran a second simulation with a different number of nodes to see how well it worked. We used the average time of 100 (or 50) rounds of block generation as the simulation results, but we did not t lose generality with this method. PoW was the most efficient, as shown in Figure 14. The performance of the proposed framework was better when random honest miners were chosen from the group of miners. Our simulation results were based on the average time of 200 rounds of block creation. This way, we did not t lose the generality of our results. There is a group of people who work together to make PoN more efficient, as shown in Figure 15. The development of concurrent multi-blocks can help. In tests, it was found that the proposed strategy was both effective and efficient at providing secure data sharing and access control for the IoT-based supply chain system. The proposed method stopped some bad things from happening, such as impersonation and Sybil attacks. People could join a federation without permission, and the process was completely fair. It allowed anyone who was a validator to become involved in a mine for a specific reward, which may change over time. If you look at Figure 16, you can see that our proposed framework increased security and significantly reduced the. To test the efficiency with one block creation, we ran a second simulation with a different number of nodes. We used the average time of 100 (or 50) rounds of block generation as the simulation results without losing generality. PoW had the highest efficiency, as demonstrated in Figure 14. The performance of the proposed framework was enhanced by generating random honest miners from the miner’s group. We used the average time of 200 rounds of block creation as the simulation result without losing generality. With the help of the mining team, the development of concurrent multi-blocks can enhance the efficiency of PoN, as shown in Figure 15. The experimental results show that the proposed strategy is effective and efficient in providing secure data sharing and access control services for the IoT-based supply chain system. The proposed methodology resisted some malicious activities, such as impersonation and Sybil attacks. The process of joining a federation was fully decentralized, permissionless, and fair. It enabled any validator to engage in a mine for explicit reward sharing, which may change dynamically. Our proposed framework improved security and significantly reduced the computational resources needed to maintain a strict level of safety security in the blockchain, as shown in Figure 16.

**Figure 17:**
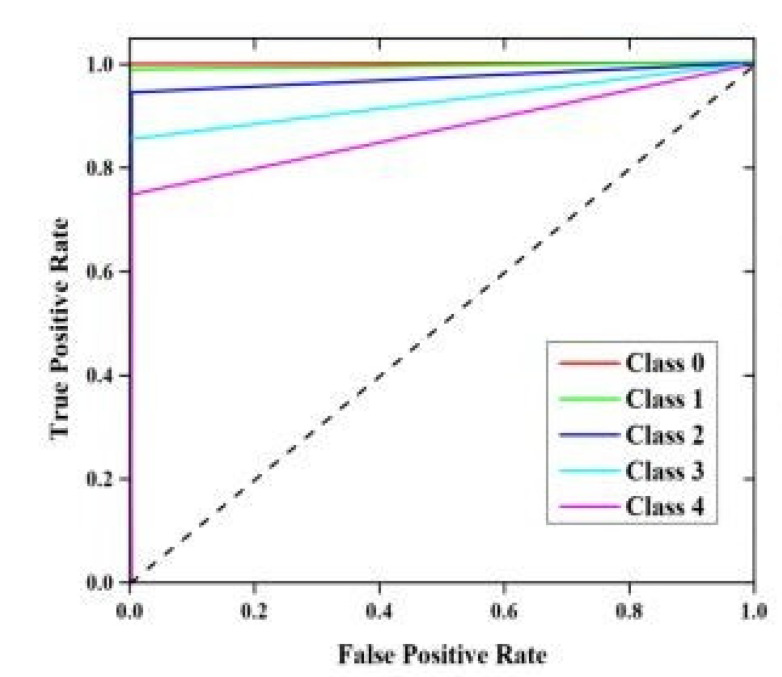
Simulation results based on the execution time and the cache hit rate.

Figure 17 represents the simulation results based on the true positive rate and the false positive rate. We classified the IoT data into different classes based on TP and TN.

**Figure 18:**
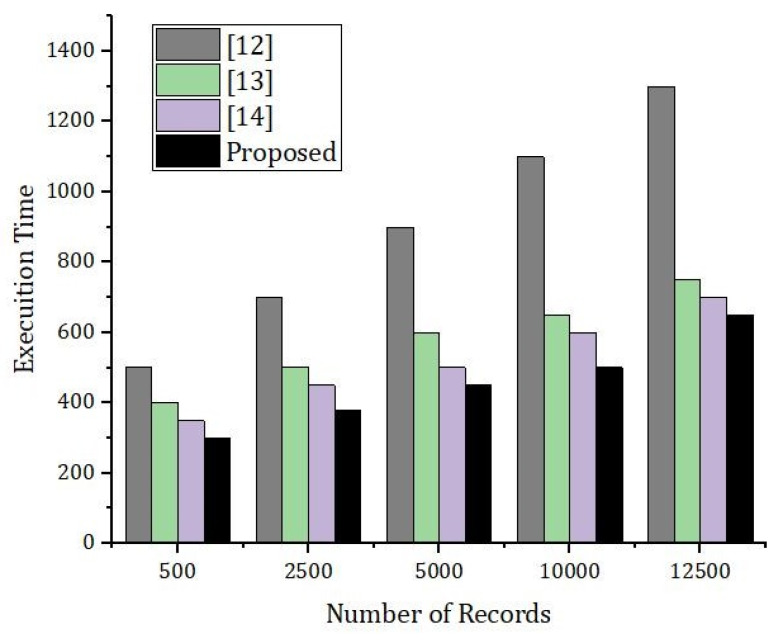
Simulation results based on the execution time and the cache hit rate.

Figure 18 represents the simulation results based on the number of records and the execution time in seconds. The number of records were counted from 500 to 10,000, respectively. We compared our proposed framework with the benchmark model. From Figure 18 it is very clear that the execution time for the same number of rounds in case of our proposed framework was much less compared to the benchmark model.

**Figure 19:**
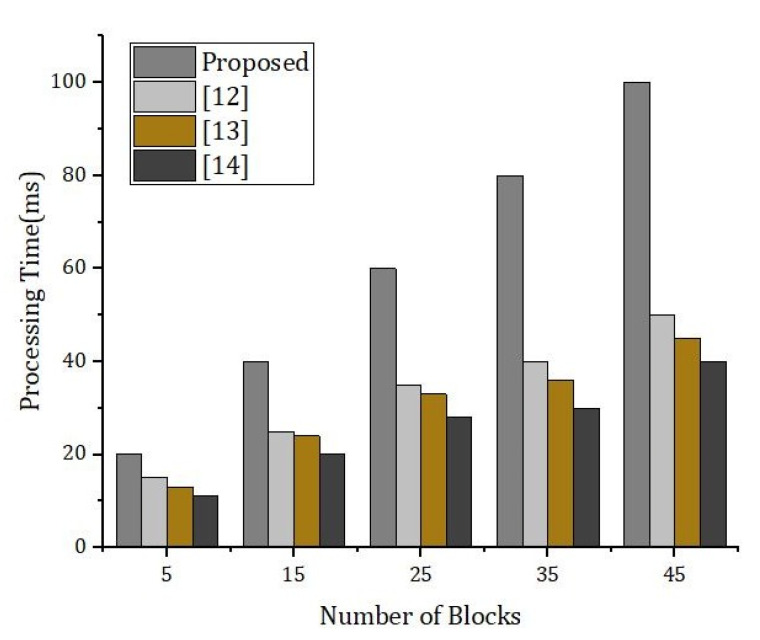
Simulation results based on the execution time and the cache hit rate.

Figure 19 describes the simulation results based on the number of blocks and the processing time. We compared our proposed framework with the benchmark models. The performance achieved by our proposed framework was better than the benchmark models.

### Security Analysis

Registration against harmful actions is required for IoT nodes. Algorithms 1 and 2 illustrate how long it takes various IoT nodes to register. As can be seen in Figure 4, the real upload time for IoT sensor data with IPFS protected storage and the number of transactions is shown. As the number of transactions rises, so does the upload time. Block mining time, block creation time, and block access time are shown in Figure 5 and Figure 6. As might be predicted, as the number of IoT sensor nodes grows, so does the elapsed time. The system design and suggested topology are shown in Figure 5 and Figure 6. In the proposed system, the transaction sign guarantees non-repudiation. Figure 5 and Figure 6 show our suggested system’s security and access control module.

## 5. Discussion and Analysis

Our proposed algorithms do not require internal or external values but provide security in the blockchain via the peer nodes. After the federation of nodes in the blockchain, a selected group of nodes manage the blockchain data, even when all the nodes are participating in blockchain, but it seems to be secure and centralized. We suggested a blockchain-based fog consensus protocol (BFCP) that employs a group of nodes in a federated capacity to provide strong security to the Blockchain. Our proposed (BFCP) Protocol is perfect for securing a blockchain because its primary token has no value at all. The proposed BFCP is ideal for blockchain security since its principal token has no monetary value. BFCP supports and provides an efficient block proposer selection and validation mechanism to find an alternate way for the mining process in PoW. This eliminates the need to solve a difficult cryptographic challenge, allowing us to significantly reduce energy usage while improving throughput. Consequently, in terms of latency and throughput, the PoIP network is more scalable than the existing consensus.

### Security Analysis and Computation Overhead Analysis

In this section, we will check and compare our proposed SML and CPBED model with comparable prototypes for storage overheads incurred by P *G_i_*, P *G_j_*, and TA. In the proposed scheme, every P Gi IoMT network stores the identities of vicinity devices in their memory table such as P GID, P G, P G, GP, and TA, respectively. In contrast, the comparative schemes store these IDs and authentication parameters in a centralized or concerned trusted authority. During the session initiation and establishment, these parameters mostly matched in the centralized TA, or concerned TA, which created storage overhead in these memory constraint devices, because they have to process each request via hop count communication or a centralized location. As a result, the performance of these authentication schemes with communication metrics was degraded.

## 6. Conclusions

Blockchain, IoMT (fog-BC-IoMT), and FC technologies all utilized the proposed architecture (fog-IoMT). To record the transactions, the BC was utilized to create a legal public, hyperdistributed EMR. Several IoMT-NODES were utilized in the testing and implementation of the architecture. The outcomes were estimated satisfactorily. This study suggested architecture for preventing data fraud by converting existing centralized database systems to block-based distributed databases. It divided the system into four parts: Cloud, fog, blockchain, and IoMT. The IoMT system was self-contained. We also looked into whether the Network Convention method could assist with public Cloud resources more effectively. HE-based encryption has been used in many research works due to its significant privacy benefits. The rapidly increasing need to ensure the privacy of data while using DL techniques makes HE very vital. In this paper, we proposed the use of the PHE-based BFCP protocol to tackle the problem of the privacy of sensitive healthcare data when using DL algorithms. The proposed protocol enabled the data to be secure and preserved a good classification accuracy. The experiments conducted on the real IoT dataset demonstrated an accuracy of 94.2% for simple EMR and 93.3% for encrypted EMR. In this sense, the proposed research can serve as a tool to classify encrypted data by non-trustworthy third parties without disclosing confidentiality. However, the main limitation of HE encryption is its slow computation, which remains the major problem of this technique. Further studies should focus on proposing hybrid encryption techniques such as FHE encryption and SSC that can be used in conjunction with one another.

## Figures and Tables

**Table 1:** Access control type, scope, scale, privacy issues, real time dataset used and the accuracy of various occupancy techniques.

Technique/Technology	Reference	Scope (Shape/Size)	Scale (Number of People)	Privacy Issues Sampling	Time	Accuracy
Access Control	[1]	NA	18	Yes	Yes	80%
	[2]	60	NA	Yes	Yes	80%
	[3]	250	NA	Yes	Yes	80%
	[4]	100	NA	Yes	NA	92%
	[5]	100	8	Yes	Yes	NA
Access Control Types	[6]	50	1	No	Yes	NA
	[7]	NA	1	No	NA	NA
	[8]	NA	14	No	Yes	86%
	[9]	NA	1	No	Yes	75%
Framework	[10]	100	2	No	Yes	NA
	[11]	NA	150	No	NA	90%
Security	[12]	50	1	Yes	Yes	93%
	[13]	200	NA	Yes	Yes	79%
	[14]	100	NA	Yes	No	NA
	[15]	50	6	Yes	No	60%
	[16]	100	30	Yes	NA	91%
Data Storage	[17]	NA	45	Yes	Yes	70%
	[18]	NA	4	No	Yes	80%
	[19]	100	4	No	Yes	NA
	[20]	40	9	No	Yes	80%
	[21]	200	23	No	NA	NA
	[22]	100	1	No	NA	70%
	[23]	50	3	No	Yes	80%
	[24]	150	3	No	Yes	NA
	[25]	NA	3	No	Yes	73%
Efficiency	[26]	NA	72	No	NA	55%
	[27]	100	41	No	No	86%
	[28]	200	10	No	No	NA
	[29]	NA	NA	No	No	91%

## Data Availability

The data used within the research can be provided by the first author upon request.
